# Low cardiorespiratory fitness in people at risk for type 2 diabetes: early marker for insulin resistance

**DOI:** 10.1186/1758-5996-1-8

**Published:** 2009-09-21

**Authors:** Silmara AO Leite, Arlene M Monk, Paul A Upham, Antonio R Chacra, Richard M Bergenstal

**Affiliations:** 1 Universidade Positivo, Curitiba, PR, Brasil; 2 Universidade Federal de São Paulo, SP, Brasil; 3 International Diabetes Center, Minneapolis, MN, USA

## Abstract

**Purpose:**

There is a significant association between insulin resistance and low cardiorespiratory fitness in nondiabetic subjects. In a population with risk factors for type 2 diabetes (T2DM), before they are insulin resistant, we investigated low exercise capacity (VO2max) as an early marker of impaired insulin sensitivity in order to determine earlier interventions to prevent development of insulin resistance syndrome (IRS) and T2DM.

**Methods:**

Cross-sectional analyses of data on 369 (78 men and 291 women) people at risk for IRS and T2DM, aged 45.6 +/- 10 years (20-65 years) old from the Community Diabetes Prevention Project in Minnesota were carried out. The cardiorespiratory fitness (VO2max) by respiratory gas exchange and bicycle ergometer were measured in our at risk non insulin resistant population and compared with a control group living in the same geographic area. Both groups were equally sedentary, matched for age, gender and BMI.

**Results:**

The most prevalent abnormality in the study population was markedly low VO2max when compared with general work site screening control group, (n = 177; 137F; 40 M, mean age 40 ± 11 years; BMI = 27.8 ± 6.1 kg/m^2^). Individuals at risk for IRS and T2DM had a VO2max (22 ± 6 ml/kg/min) 15% lower than the control group VO2max (26 ± 9 ml/kg/min) (p < 0.001). It was foun that VO_2_max was inversely correlated with HOMA-IR (r = -0.30, p < 0.0001).

**Conclusions:**

Decreased VO2max is correlated with impaired insulin sensitivity and was the most prevalent abnormality in a population at risk for IRS and T2DM but without overt disease. This raises the possibility that decreased VO2 max is among the earliest indicators of IRS and T2DM therefore, an important risk factor for disease progression.

## Introduction

The global prevalence of diabetes in 2009 among adults ≥ 20 years of age is estimated to be 171 million. This figure is 11% higher than the previous estimate [1] and the projection for year 2030 maybe considerably higher than the current estimate[2]. Diabetes is a devastating and costly public health problem. In addition to the serious impact on quality of life, diabetes can lead to accelerated arterial aging and potentially decreased life expectancy. Endothelial dysfunction is a key abnormality found in insulin-resistance states and vascular dysfunction [3]. Exercise capacity (VO2max) is strongly correlated with insulin sensitivity [4] and endothelial dysfunction [5].

Traditional and nontraditional cardiovascular (CVD) risk factors associated with metabolic syndrome are present long before the onset of clinical diabetes, which raises the question about their usefulness in assessing risk of developing diabetes and CVD [6]. Increasing evidence that interventions involving changes in diet and physical activity or pharmacological treatment can prevent/delay type 2 diabetes provides an impetus for wider implementation of preventive approaches [7]. An early marker for insulin resistance syndrome and diabetes may identify individuals in whom earlier interventions may improve arterial stiffness before significant endothelial dysfunction.

As part of the Community Diabetes Prevention Project (CDPP) obesity, HbA1c, fasting insulin level, LDL-cholesterol, HDL-cholesterol, triglyceride, HOMA-IR, fasting plasma glucose, microalbumin, waist/hip ratio and VO2max were investigated as early markers of impaired glucose regulation. This study included individuals who were at risk for T2DM but who had not yet developed the full blown insulin resistance syndrome (hypertension, hyperinsulinemia, obesity, IGT, and dyslipidemia) or T2DM at baseline.

## Methods

### Study Population

To identify those at risk for eventually developing IRS and/or T2DM, a risk assessment tool was developed [8-11]. The assessment tool includes questions from the American Diabetes Association diabetes screening questionnaire [12] (family history of diabetes, more than 20% over maximum ideal weights, history of diabetes during pregnancy or having a baby over 9 pounds, sedentary lifestyle) and additional questions related to IRS (hypertension, dyslipidemia and race/ethnicity). This risk assessment tool was distributed to the community through the mail, clinics, hospital and pharmacies, and through local television and public radio by request.

Subjects were included if they met the following criteria: age 20 to 65 years and family history of diabetes or history of gestational diabetes, and/or presence of one to three risk factors for T2DM (obesity, hypertension, dyslipidemia) but without all the major components of IRS. Subjects were excluded if they reported having no risk factors or either limited exercise alone or only racial/ethnicity alone as the only risk factor, or had a severe medical condition that would make it unlikely that they could perform an exercise evaluation or complete the five-year study. In addition those who were found to have type 2 diabetes and fasting blood glucose > = 110, were excluded from this cross-section analysis.

The control group was Highsmith employees matched for age, gender, level of activity and BMI, whose VO2 max was measured with same Cardio2 Bike protocol as part of annually health assessment (table 1).

**Table 1:** Characteristics of external comparison group and people at risk for type 2 diabetes and/or IRS (CDPP)

VARIABLE	CONTROL	CDPP group
N	177	369

Age (years)	40 ± 11	45.7 ± 10

Gender (females/males)	137/40	291/78

BMI (kg/m^2^)	27.8 ± 6.1	28.8 ± 6.3

### Analysis of oxygen consumption (VO2max)

Cardiorespiratory fitness was measured directly from respiratory gas exchange during a maximal, symptom-limited exercise tolerance test on a bicycle ergometer (Cardio2 Bike from Medgraphics, St Paul, MN). The resulting VO_2_max value in ml of O_2 _per kg per minute allows a direct comparison of individuals regardless of body weight. Individuals' scores are categorized by age (grouped by decade) and gender as either healthy, moderate risk or high risk [13].

### Physical and Laboratory assessment

Physical assessment evaluated height and weight (for calculation of Body Mass Index), waist/hip ratio, blood pressure (sitting), and fitness level (VO2max). For all subjects fasting plasma glucose was measured and those with FPG ≥ 110 received a 75 g OGTT. In addition, HbA1c (assay standardized according to DCCT trial), lipid fractionation (total cholesterol, HDL-cholesterol, triglycerides and calculated LDL-cholesterol), serum fasting insulin levels, serum C-peptide and microalbumin were obtained.

The calculation of HOMA [14] was performed with the following formula:

### Statistical Analysis

The "Student t-test of significance" and "One-way Analysis of Variance (ANOVA)"was used to test differences of VO_2_max between CDPP group with external control group, being performed at the 0,05 level of significance. VO_2_max, deciles were determined separately for males and females.

To determine the association of VO_2_max levels and insulin sensitivity, The population at risk was stratified according to fasting plasma glucose to evaluate the VO2max correlation from normal to impaired glucose regulation in three groups: Fasting plasma glucose (FPG) under 100 mg/dl, FPG = 101-109, FPG> = 110.

## Results

1769 people completed and returned the CDPP risk assessment tool to join "at risk registry", of whom, 1423 had at least one positive risk factor (excluding sedentary lifestyle or ethnicity as the only risk factor). Of the 466 who signed the informed consent to participate in the project 28 dropped voluntarily before completing baseline screening, diabetes was discovered and diagnosed in 14 individuals and 8 were found to have the full IRS, the remaining 418 at risk non insulin resistant individuals were followed for 5 years. Table 2 represents the population stratified according to ADA criteria for impaired fasting plasma glucose, considering FPG under 100 mg/dl, FPG = 101 to 109 and FPG> = 110. There was no significant difference in VO2max between these groups. The analysis was limited to the 369 subjects with fasting plasma glucose under 110 mg/dl, following WHO criteria to exclude impaired glucose regulation.

**Table 2:** Stratification VO2max value and Fasting Plasma Glucose levels

	VO2 max (ml/kg/min)		
FPG (mg/dl)	Means*	N	SD
<100	22,28	246	6,02
100-109	21,29	121	5,97
> = 110	20,18	49	6,28
All Groups	21,74	416	6,06

*p-value = 0.054

The results of physical and laboratory assessment are shown in table 3. Table 4 lists the prevalence of risk factors in this population. VO2max was the most prevalent abnormality in this population of individuals who were at risk for type 2 diabetes but who did not have IRS or T2DM at baseline. VO_2_max had an inverse correlation with HOMA-IR(r = -0.30, p < 0.0001), showing that increased insulin resistance is associated with decreased VO_2_max.

**Table 3:** Results of physical and laboratory evaluation of CDPP group (n = 369)

Variables	Results (mean ± SD)
BMI (kg/m^2^)	28.8 ± 6.3

Cholesterol (mg/dl)	206 ± 38.5

LDL-C (mg/dl)	128 ± 33

HDL-C (mg/dl)	49 ± 15

Triglycerides (mg/dl)	154 ± 90

Fasting Glucose (mg/dl)	96 ± 6.7

Systolic BP/Diastolic BP (mm/Hg)	125 ± 14.5/80 ± 8.4

Waist/Hip Ratio - Fem	0.81 ± 7

Waist/Hip Ratio - Male	0.92 ± 6

VO2max (ml/kg/min)	21.9 ± 6

Insulin (μU/ml)	7.2 ± 5.1

C-Peptideo (ng/ml)	2.4 ± 2.0

HbA1c (%)	5.4 ± 0.5

HOMA score	1.51 ± 1.13

**Table 4:** Prevalence of risk factors in this population.

Measure	Value	% Subjects (N = 369)
FBG	> = 100	41%

SBP	> = 130	38%

DBP	> = 85	28%

Cholesterol	> = 200	59%

LDL	> = 130 > = 100	46% 79%

HDL	M<40, F<50	53%

Triglycerides	>150	42%

BMI	> = 30	34%

A1C	>5.8	9%

Fasting Insulin	>23	3%

C-peptide	>4.0	7%

WHR	M> = 1.00 or F> = 0.85	24%

VO2 Max based on age and gender	High Risk M<33, F<26 Moderate to High Risk	84% 96%

Positive Family History		70%

96% of individuals at risk for type 2 diabetes had VO2 max results under health range for their age and sex of whom 11% were in moderate risk and 85% were in high risk for developing disease related with low aerobic capacity (diabetes, stroke, coronary disease and some forms of cancer) (24).

The control group had a VO_2_max = 26 ± 9 ml/kg/min 15% higher than CDPP population (p < 0.001). Cardiorespiratory fitness was considered healthy in 14% of control group, compared with only 4% of CDPP group (x^2 ^= 11.4 p < 0.001) shown at table 5.

**Table 5:** CDPP and External Control Group Comparison

Group	Healthy VO2max(ml/kg/min) M>38; F>30	Moderate Risk VO2max(ml/kg/min) M = 33-38; F = 26-30	High Risk VO2max(ml/kg/min) M<33; F<26
Control	14%	13%	73%

CDPP	4%	12%	84%

X^2 ^= 11.4 (p < 0.001)

## Discussion

In a population at high risk for IRS and T2DM, individuals were enrolled at an earlier stage in the progression of disease when compared with other prevention studies. Importantly, low exercise capacity or cardiorespiratory fitness (VO2max) was the most prevalent abnormality at baseline assessment for this population. When compared a control group with similar anthropometric measures, as well as low level of physical activity, both groups had marked low maximal oxygen consumption however, on average VO2max was 15% higher in the control subjects. This finding is in line with several recent studies that showed significant association between insulin resistance and low physical fitness in nondiabetic subjects [15,16]. In addition, sedentary lifestyle and low cardiorespiratory fitness have been associated with increased risk for T2DM [17-19].

Individuals with type 2 diabetes, have previously been shown to have 20% lower cardiorespiratory fitness (VO2max) compared to control subjects [20]. People at risk may progressively decrease aerobic capacity in the natural history of the development of insulin resistance syndrome and T2DM.

Wei et al [21] found that low cardiorespiratory fitness is significantly associated with impaired fasting glucose and type 2 diabetes, as well as independent predictor of all-cause mortality in men with T2DM [22].

Eriksson and Lindgarde showed a progressive decrease at VO2max as glucose regulation declines from normal to IGT and T2DM [23]. With respect to this progression, individuals in our population fell in the normal to IGT range for blood glucose, while the majority (84%) had markedly low VO2 max levels, which is an early marker of decreased insulin sensitivity.

An early step in the development of insulin resistance in offspring of patients with type 2 diabetes is impaired mitochondrial activity [24]. Kaplan et al [25] concluded that insulin plays an important role in the regulation of mitochondrial anion transporter function during the Krebs cycle Oxygen consumption for oxidation of Acetyl-CoA produced by glycolysis.

This abnormality could be the earlier impairment of insulin sensitivity in the study population, since 70% of them had family history of diabetes. Low number of mitochondria, genetically determined, explains the altered consumption of glucose and oxygen which may impact an individual's ability to achieve an acceptable fitness level and consequently have low VO2max.

Our study has limitations. The cross-section design does not allow establishing cause-effect relationship. Also, HOMA IR is not an ideal way to measure insulin resistance and we have not calculated the control group's HOMA IR index. In spite of that, the low HOMA IR level in the study population showed that they were not insulin resistant yet. However, they had already presented markedly low VO2max, which is related to endothelium dysfunction. This raises the possibility that decreased VO2 max is among the earliest indicators of IRS and T2DM therefore, an important risk factor for disease progression.

Previous studies have suggested that higher levels of regular physical activity and cardiorespiratory fitness (VO2max) are associated with a reduced risk of coronary heart disease [26,27]. Low physical fitness has been associated with increased clustering of the metabolic abnormalities associated with the metabolic syndrome [28,29]. Ferreira et al found that VO2max was inversely associated with arterial stiffness, and this was independent of the metabolic syndrome [30].

Physical activity levels are currently overestimated particularly in the obese population. The finding of decreased VO2max as an early marker for IRS/T2DM may encourage people at risk to participate in regular physical activity and improve cardiorespiratory fitness in order to prevent insulin resistance, type 2 diabetes and atherosclerosis acceleration.

## Competing interests

The authors declare that they have no competing interests.

## Authors' contributions

SAOL conceived of the study, participated in the collect data, write the manuscript, AMM participated in the collect data and coordination, PAU participated in the design of the study and performed the statistical analysis, RMB participated in the design of the study and is the Senior of the project. All authors read and approved the final manuscript.
